# HIV positive patient with GBS-like syndrome

**DOI:** 10.1099/jmmcr.0.005107

**Published:** 2017-09-01

**Authors:** Samantha J. Shepherd, Heather Black, Emma C. Thomson, Rory N. Gunson

**Affiliations:** ^1^​ West of Scotland Specialist Virology Centre, Level 5, New Lister Building, 10-16 Alexandra Parade, Glasgow G31 2ER, UK; ^2^​ Infectious Diseases Unit, Queen Elizabeth University Hospital, 1345 Govan Road, Glasgow, G51 4TF, UK; ^3^​ MRC-University of Glasgow Centre for Virus Research, Stoker Building, 464 Bearsden Road, Glasgow G61 1QH, UK

**Keywords:** guillain-barre syndrome, HIV, polyneuropathy

## Abstract

**Introduction.** Guillain–Barré Syndrome (GBS) is an acute demyelinating polyneuropathy which can occur post-infection. Criteria of diagnosis of GBS include areflexia with progressive bilateral weakness in arms and legs. GBS can lead to severe respiratory and cardiac complications. The fatality rate can be up to 5 % in patients, depending on the severity of the symptoms. HIV can cause a range of neurological disorders including, on rare occasions, GBS. GBS can occur at any stage of HIV infection, highlighting the complexity of diagnosis of GBS within HIV patients.

**Case presentation.** A 57 year old female with lumbar back pain radiating to the legs, poor mobility and tiredness, with reports of a viral-like illness four days previously, was initially diagnosed with a lower respiratory tract infection and discharged. Seventeen days later the patient was readmitted to hospital with progressive lower and upper limb weakness, areflexia and sensory loss. She was diagnosed with GBS and was unexpectedly discovered to be HIV-positive. HIV avidity was low indicating a recently acquired HIV infection. The patient was treated with intravenous immunoglobulin for five days for the GBS and commenced antriretrovirals for HIV. The patient was discharge from hospital 53 days after admission with walking aids and regular physiotherapy follow-up.

**Conclusion:**

. This case highlighted the need for all clinicians to be aware that patients with symptoms of GBS, regardless of clinical history should be offered an HIV test. GBS can be the first sign a patient is HIV-positive.

## Abbreviations

CNS, central nervous system; CT, computerised tomography; EBV, Epstein Barr virus; GBS, Guillain-Barre syndrome; HHV6, human herpes virus 6; HSV, herpes simplex virus; LRTI, lower respiratory tract infection; VZV, varicella zoster virus; WBC, white blood cell.

## Introduction

Guillain–Barré syndrome (GBS) is an acute inflammatory demyelinating polyneuropathy which has an incidence rate in Europe of less than 2 cases per 100 000 [1]. It is an autoimmune disease which often occurs post-infection. Sixty percent of patients recall gastroenteritis or a respiratory infection within 6 weeks prior to GBS symptoms [1]. Diagnostic criteria include areflexia with progressive bilateral weakness in the arms and legs [2] and this can be complicated by severe respiratory and cardiac complications. The fatality rate is 5 and 20 % develop long term neurological sequelae [3]. Several viral infections have been associated with GBS including cytomegalovirus (CMV), Hepatitis E virus (HEV) and Zika virus [4–6]. GBS can also occur with both acute and long-standing HIV infections [7]. The British HIV Association (BHIVA) guidelines state that a patient presenting with GBS should be screened for HIV [8].

The case presented highlights a patient who was admitted to hospital with pain and bilateral limb weakness and who was discharged five days later without virological screening. It was on re-admission to hospital with progressive symptoms that the patient was found to be HIV-positive. This case highlights the importance of considering viral diagnoses, including HIV, in patients with GBS, including those with no apparent blood-borne virus risk factors.

## Case report

A 57 year old female was admitted to hospital with lumbar back pain radiating to the legs, poor mobility and tiredness. She reported a viral-like illness four days prior to admission characterised by mild cough, diarrhoea and vomiting. Blood tests indicated: C-reactive protein 19 mg l^−1^, sodium level 120 mmol l^−1^, white blood cell count 12.1×10^9^ l^−1^ and platelet count 387×10^9^ l^−1^. A CT scan of brain, chest, abdomen and pelvis revealed pulmonary nodules likely to be inflammatory or infective in nature and was otherwise unremarkable. The initial diagnosis was lower respiratory tract infection (LRTI) with hyponatraemia. No neurological abnormalities were documented and muscle weakness was attributed to infection and electrolyte imbalance. The differential diagnosis of hyponatraemia was attributed to LRTI or eplerenone which the patient had been prescribed following a myocardial infarction 9 months previously. Due to a history of alcohol excess, the patient was started on Pabrinex and amoxicillin and the eplerenone was discontinued. Her sodium level improved and she was discharged 5 days later with a follow-up appointment.

Seventeen days later the patient was re-admitted to hospital with progressive lower limb weakness, new hand weakness and sensory loss in the hands and feet. On examination there was evidence of reduced power in the upper and lower limbs with areflexia and sensory loss in a glove and stocking distribution.

## Investigations

A lumbar puncture revealed a raised protein [1.69 g l^−1^ (range 0.10–0.50 g l^−1^)], pleocytosis [white blood cell count (WBC) was 39 cells mm^−^^3^ with 90 % lymphocytes and 10 % polymorphs] and low glucose [2.4 (range 2.5–4.5 mmol l^−1^)]. Serum and cerebrospinal fluid (CSF) revealed evidence of oligoclonal bands.

Nerve conduction studies showed mixed sensory motor polyneuropathy with ongoing axonal loss affecting both the upper and lower limb muscles. There were no demyelinating features. The ganglioside antibody screen was normal. An MRI brain showed small vessel disease only.

Virological investigations found the patient to be HIV-1 positive by the Abbott Architect Ab/Ag combination assay, BioMeriuex miniVidas and the Bio-Rad Geenius reader HIV 1/2 conformational test. The HIV viral load using the Abbott RealTi*m*e HIV-1 was 195 328 copies ml^−1^. Using an in-house avidity assay the HIV avidity was low (20 %) indicating that the patient had a recent infection within the past three to four months [9]. The CD4 count was 730 cells mm^−^^3^. The patient was HBV surface antigen-, HBV core IgG-, HCV antibody- and syphilis-negative. Toxoplasma IgG was equivocal and IgM was negative. There was no evidence of *Salmonella* species, *Shigella*, *E.coli* O157, *Campylobacter*, *Clostridium difficle* or *Cryptosporidium* oocysts in faecal samples. The cerebrospinal fluid was negative by PCR for HSV1, HSV2, VZV, enterovirus, parechovirus, JC polyomavirus, CMV, EBV, HHV6, meningococcus, pneumococcus and *Haemophilus influenzae*. The patient was negative for *Cryptococcus neoformans* in both the CSF and blood. Using an in-house real-time PCR assay, the CSF was found to be HIV RNA-positive. The HIV RNA *pol* gene was sequenced and found to be a complex recombinant form (GenBank accession number MF372642).

## Diagnosis

The final diagnosis was HIV seroconversion illness with bilateral sensory and motor axonal neuropathy of the upper and lower limbs. As the low HIV avidity result, CD4 count and clinical manifestations indicated that the patient had been recently infected with HIV, and the only risk was sexual contact with her long-term partner (who had spent long periods of time working in sub-Saharan Africa), he was tested for HIV and found to be HIV-1 positive with a CD4 count 144 cells mm^−^^3^ and HIV viral load of 208 000 copies ml^−1^. The HIV avidity result was high (99 %) indicating a long-standing infection. Based on the HIV *pol* gene, the HIV subtype was found to be a complex recombinant form (GenBank accession number MF372643). Although both patient and partner had complex recombinant forms of HIV, when the sequences were aligned there were only five main base pair differences between the two sequences (Fig. S1, available in the online Supplementary Material).

## Treatment

The patient was commenced on intravenous immunoglobulins (IVIG) at 400 mg kg^−1^ day^−1^ for 5 days and started on Triumeq, a once daily tablet combination of the antiretrovirals abacavir, lamivudine and dolutegravir. The HIV viral load dropped to <40 detected copies ml^−1^ two months after therapy was commenced.

## Outcome and follow-up

The patient was discharged from hospital 53 days later with improved mobility but still requiring the use of walking aids. The patient continues to be compliant with her antiretrovirals and is now attending regular physiotherapy and outpatient clinic follow up.

## Discussion

HIV can cause a range of neurological disorders affecting cognitive function and the peripheral nervous system [10–12]. Patients with GBS need to be offered testing for HIV as this syndrome can often be the first sign a patient is HIV-positive [13, 14]. Clinical signs of GBS are similar for patients regardless of their HIV status [13, 15]. Lower limb paraesthesia and bilateral lower limb weakness with areflexia have been reported in HIV-positive patients with GBS [16–20]. Flu-like symptoms, fever, maculopapular rash and gastrointestinal symptoms prior to onset of GBS have been reported in some of these patients [16, 17, 19]. The variety of symptoms reported with GBS following HIV seroconversion, highlight its complexity. Quadriparesis occurred in one patient, with improvement following IVIG treatment [18]. In two other reported cases, one of which was fatal, both patients progressed to weakness in all limbs as well as dyspnoea, dysarthria and facial palsy [16, 17]. GBS has also been documented in chronic HIV infection [19, 20]. In one chronic case the patient had stopped antiretroviral therapy three months before limb weakness began [20]. Another patient with chronic HIV, was reported to have GBS symptoms two months after commencement of antiretroviral therapy [19].

Molecular mimicry and antiganglioside antibodies are considered to be involved in the pathogenesis of GBS following infection [21]. The history, examination and clinical disease course of HIV-associated GBS resembles non-HIV-related GBS. Nerve conduction studies reveal similar patterns to those of HIV-negative patients. Elevated CSF protein is found in GBS of any cause, while WBC pleocytosis is seen more frequently in HIV-positive patients [14]. The patient in this report had both elevated protein and WBC pleocytosis in her CSF.

HIV virus was detected in the CSF fluid of our patient. Central nervous system (CNS) inflammation and detection of HIV RNA in the CSF can occur in the early stages of acute infection [22–24]. HIV can have both direct and indirect neurotoxic effects on the CNS and peripheral nervous system [25–27]. HIV can continue to maintain a presence in the CNS even under antiretroviral therapy [28, 29]. The patient in this case was commenced on abacavir, lamivudine and dolutegravir. All three of these antiretrovirals have been shown to effectively reduce HIV RNA levels in the CSF [30, 31].

A higher frequency of GBS has been found in African HIV-positive patients [19]. The partner of this particular patient had been working in Africa and had been engaged in high-risk sexual activity. The HIV avidity result for the partner indicated a chronic infection which, given the recombinant HIV subtype, is likely to have been acquired in Africa.

There is insufficient evidence on long-term outcomes in HIV-positive GBS patients. It has been suggested that compared with HIV-negative individuals HIV-related GBS may be related to more frequent recurrent episodes [18].

In some circumstances avidity assays can generate false recent results [32]. This can occur in HIV avidity assays when the HIV viral load is less than 1000 copies ml^−1^, the patient CD4 count is less than 250 cells mm^−^^3^ or the patient is on antiretroviral therapy prior to avidity testing [33]. The patient in this case did not have any factors that would contribute to a false low level avidity result. This is the first case, to our knowledge, where a recent HIV infection has been confirmed by HIV avidity in a patient with GBS.

This patient did not consider herself to be at high risk for HIV infection. Patients with GBS should be tested for HIV and this syndrome can often be the first sign a patient is HIV-positive [18, 20]. This case highlights the importance of testing all patients with GBS-like and neurological symptoms for HIV regardless of risk factors.

## Supplementary Data

174 Supplementary File 1
