# Supplementary material for: HIV positive patient with GBS-like syndrome
Source: JMM Case Rep. 2017 Sep 1;4(8):e005107. doi: 10.1099/jmmcr.0.005107 (PMC5610709; doi:10.1099/jmmcr.0.005107)
Supplement: Supplementary File 1 [file jmmcr-4-5107-s001.pdf]

Figure: Sequence alignment of the HIV pol gene region for both the patient (HIVPt1, GenBank accession number MF372642) and the partner of the patient (HIVPt2, GenBank accession number MF372643). The boxed areas indicate the 5 nucleotide base differences between the two sequences. Mixed nucleotide bases are represented as R (G or A) and Y (C or T).

```
                 10         20         30         40         50         60         70         80         90        100
HIVPt1  CCCTCAAATC ACTCTTTGGC AGCGACCCGT AGTTACAGTA CAGATAGAGG GACAGTTAAG AGAAGCTCTA TTAGATACAG GAGCAGATGA TACAGTATTA
HIVPt2  CCCTCAAATC ACTCTTTGGC AGCGACCCRT AGTTACAGTA CAGATAGAGG GACAGTTAAG AGAAGCTCTA TTAGATACAG GAGCAGATGA TACAGTATTA

                110        120        130        140        150        160        170        180        190        200
HIVPt1  GAAGACATAA ATTTGCAAGG AAAATGGAAA CCAAAAATGA TAGGGGGAAT TGGAGGTTTT ATCAAAGTAA GGGAGTATGA GCAAGTAGTT GTAAATATCT
HIVPt2  GAAGAYATAA ATTTGCAAGG AAAATGGAAA CCAAAAATGA TAGGGGGAAT TGGAGGTTTT ATCAAAGTAA GGGAGTATGA GCAAGTAGTT GTAAATATCT

                210        220        230        240        250        260        270        280        290        300
HIVPt1  GTGGAAAAAA GGCCATAGGT ACAGTATTGG TAGGACCTAC ACCTGTCAAT ATAATTGGAA GGAATTTATT GACTCAGATT GGTTGCACTC TAAATTTCCC
HIVPt2  GTGGAAAAAA GGCCATAGGT ACAGTATTGG TAGGACCTAC ACCTGTCAAT ATAATTGGAA GGAATTTATT GACTCAGATT GGTTGCACTC TAAATTTCCC

                310        320        330        340        350        360        370        380        390        400
HIVPt1  AATTAGTCCT ATTGAAACTG TACCAGTGAA ATTAAAGCCA GGAATGGATG GCCCAAAGGT TAAACAATGG CCATTAACAG AAGAAAAAAT AAAGGCATTA
HIVPt2  AATTAGTCCT ATTGAAACTG TACCAGTGAA ATTAAAGCCA GGAATGGATG GCCCAAAGGT TAAACAATGG CCATTAACAG AAGAAAAAAT AATGGCATTA

                410        420        430        440        450        460        470        480        490        500
HIVPt1  ACAGAAATCT GTGCAGATAT GGAAAAGGAA GGAAAAATTT CAAAAATTGG GCCTGAGAAT CCATATAACA CACCAGTGTT TGCCATAAAG AAAAAAGATA
HIVPt2  ACAGAAATCT GYGCAGATAT GGAAAAGGAA GGAAAAATTT CAAAAATTGG GCCTGAGAAT CCATATAACA CACCAGTGTT TGCCATAAAG AAAAAAGATA

                510        520        530        540        550        560        570        580        590        600
HIVPt1  GTACTAAATG GAGAAAATTA GTTGATTTTA GGGAACTCAA TAAAAGAACT CAAGACTTCT GGGAAGTTCA ATTAGGGATA CCACATCCAG CAGGGTTGAA
HIVPt2  GTACTAAATG GAGAAAATTA GTTGATTTTA GGGAACTCAA TAAAAGAACT CAAGACTTCT GGGAAGTTCA ATTAGGGATA CCACATCCAG CAGGGTTGAA

                610        620        630        640        650        660        670        680        690        700
HIVPt1  AAAGAAAAAA TCAGTAACAG TATTAGATGT GGGGGATGCA TATTTCTCAG TCCCTCTAGA TCCAGACTTC AGGAAGTATA CTGCATTCAC TATACCTAGT
HIVPt2  AAAGAAAAAA TCAGTAACAG TATTAGATGT GGGGGATGCA TATTTCTCAG TCCCTCTAGA TCCAGACTTC AGGAAGTATA CTGCATTCAC TATACCTAGT

                710        720        730        740        750        760        770        780        790        800
HIVPt1  AGAAACAATG AGACACCAGG AGTTAGATAT CAATACAATG TGCTACCACA AGGATGGAAA GGGTCACCGG CAATATTCCA GAGTAGCATG ACAAAAATCT
HIVPt2  AGAAACAATG AGACACCAGG AGTTAGATAY CAATACAATG TGCTACCACA AGGATGGAAA GGGTCACCGG CAATATTCCA GAGTAGCATG ACAAAAATCT

                810        820        830        840        850        860        870        880        890        900
HIVPt1  TAGAACCATT TAGAAAGAAA AACCCAGAGA TAGTTATCTA TCAATACATG GATGACTTGT ATGTAGGATC TGACTTAGAA ATAGGACTAC ATAGAGCAAA
HIVPt2  TAGAACCATT TAGAAAGCAA AACCCAGAGA TAGTTATCTA TCAATACATG GATGACTTGT ATGTAGGATC TGACTTAGAA ATAGGACTAC ATAGAGCAAA

                910        920        930        940        950        960        970        980        990       1000
HIVPt1  AATAGAGGAG TTAAGAGCTC ACTTGTTGAA GTGGGGATTA ACTACACCAG ACAAAAAACA TCAGAAAGAA CCCCCATTTA TGTGGATGGG ATATGAGCTC
HIVPt2  AATAGAGGAG TTAAGAGCTC ACTTGTTGAA GTGGGGATTA ACTACACCAG ACAAAAAACA TCAGAAAGAA CCCCCATTTA TGTGGATGGG ATATGAGCTC

               1010       1020       1030       1040       1050       1060       1070       1080       1090       1100
HIVPt1  CATCCTGACA AATGGACAGT ACAACCTATA AAATTGCCTG ACAAGGAAAA CTGGACTGTC AATGATATAC AAAAATTAGT AGGAAAACTA AATTGGGCAA
HIVPt2  CATCCTGACA AATGGACAGT ACAACCTATA AAATTGCCTG ACAAGGAAAA CTGGACTGTC AATGATATAC AAAAATTAGT AGGAAAACTA AATTGGGCAA

               1110       1120       1130       1140       1150       1160       1170       1180       1190       1200
HIVPt1  GTCAAATTTA TCCAGGGATT AAAGTAAGGC AATTATGTAA ACTCCTTAGG GGGGCCAAAG CACTAACAGA CATAGTATCA CTGACTGCAG AAGCAGAATT
HIVPt2  GTCAGATTTA TCCAGGGATT AAAGTAAGGC AATTATGTAA ACTCCTTAGG GGGGCCAAAG CACTAACAGA CATAGTATCA CTGACTGCAG AAGCAGAATT

               1210       1220       1230       1240       1250       1260       1270       1280       1290       1300
HIVPt1  AGAATTGGCA GAAAACAGGG AAATTCTAAA AGACCCAGTA CATGGAGTAT ATTATGACCC ATCAAAAGAC TTAATAGCAG AAGTACAAAA ACAAGGGCTA
HIVPt2  AGAACTRGCA GAGAACAGGG AAATTCTAAA AGACCCAGTA CATGGAGTAT ATTATGACCC ATCAAAAGAC TTAATAGCAG AAGTACAAAA ACAAGGGCTA

               1310       1320       1330       1340       1350       1360       1370       1380       1390       1400
HIVPt1  GACCAATGGA CATATCAAAT ATATCAAGAG CCATTCAAGA ACCTGAAAAC AGGGAAATAT GCAAAAAGGA GGGCTGCCCA CACTAATGAT GTAAAGCAAT
HIVPt2  GAYCAATGGA CATATCAAAT ATATCAAGAG CCATTCAARA ACCTGAAAAC AGGGAAATAT GCAAAAAGGA GGGCTGCCCA CACTAATGAT GTAAAGCAAT

               1410
HIVPt1  TAACAGAAGT GGTACA
HIVPt2  TAACAGAAGT GGTACA
```
